# Recent advances in clustering methods for protein interaction networks

**DOI:** 10.1186/1471-2164-11-S3-S10

**Published:** 2010-12-01

**Authors:** Jianxin Wang, Min Li, Youping Deng, Yi Pan

**Affiliations:** 1 School of Information Science and Engineering, Central South University, Changsha 410083, China; 2 Department of Computer Science, Georgia State University, Atlanta, GA30303, USA; 3 Rush University Cancer Center, Rush University Medical Center, Chicago, IL 60612, USA

## Abstract

The increasing availability of large-scale protein-protein interaction data has made it possible to understand the basic components and organization of cell machinery from the network level. The arising challenge is how to analyze such complex interacting data to reveal the principles of cellular organization, processes and functions. Many studies have shown that clustering protein interaction network is an effective approach for identifying protein complexes or functional modules, which has become a major research topic in systems biology. In this review, recent advances in clustering methods for protein interaction networks will be presented in detail. The predictions of protein functions and interactions based on modules will be covered. Finally, the performance of different clustering methods will be compared and the directions for future research will be discussed.

## Background

Within cells, proteins seldom act as single isolated species to perform their functions. It has been observed that proteins involved in the same cellular processes often interact with each other [1]. Protein-protein interactions are thus fundamental to almost all biological processes [2]. As advances in high-throughput technologies, such as yeast-two-hybrid, mass spectrometry, and protein chip technologies, huge data sets of protein-protein interactions are available [3]. Such protein-protein interaction data can be naturally represented in the form of networks, which not only give us the initial global picture of protein interactions on a genomic scale but also help us understand the basic components and organization of cell machinery from the network level.

A protein interaction network is generally represented as an interaction graph with proteins as vertices (or nodes) and interactions as edges. Various topological properties of protein interaction networks have been studied, such as the network diameter, the distribution of vertex degree, the clustering coefficient and etc. These network analyses have shown that protein interaction networks have the features of a scale-free network [4-7] and “small-world effect” [8,9]. Beyond the discussions of the scale-free and small-world properties, an important challenge for system biology is to understand the relationship between the organization of a network and its function. It has been shown that clustering protein interaction networks is an effective approach to achieve this goal [10].

Clustering in protein interaction networks is to group the proteins into sets (clusters) which demonstrate greater similarity among proteins in the same cluster than in different clusters. In protein interaction networks, the clusters correspond to two types of modules: protein complexes and functional modules. Protein complexes are groups of proteins that interact with each other at the same time and place, forming a single multimolecular machine, such as the anaphase-promoting complex, RNA splicing and polyadenylation machinery, protein export and transport complexes, etc [11]. Functional modules consist of proteins that participate in a particular cellular process while binding each other at a different time and place, such as the yeast pheromone response pathway, MAP signaling cascades, etc [11].

Recently, many research works have been done on the problem of clustering protein interaction networks. These works rely on very different ideas and approaches. This paper tries to help readers keep up with recent and important developments in the field, and to give readers a comprehensive survey on the different approaches. This paper is organized as follows: At first, the graph-based clustering methods including the density-based and local search algorithms, the hierarchical clustering algorithms, and other optimization-based algorithms, are given in Section 2. Then the approaches of combination with other information are discussed and some ensembles are given in Section 3. In Section 4, the validation and comparison of the clustering methods are discussed. Then the application of the clustering methods for protein function prediction and protein-protein interaction prediction are given in Section 5. At last, challenges and directions for future research are discussed in Section 6.

## Graph-based clustering methods

In general, a protein interaction network is represented as an undirected graph *G*(*V,E*)*,* where vertices represent proteins and edges represent interactions. The relationship between two proteins can be the simple binary values: 1 or 0, where 1 denotes the two proteins interact and 0 denotes the two proteins do not interact. In such cases, the graph is unweighted. Sometimes, the edges of graph *G* are weighted with a value between 0 and 1. In such cases, the weight represents the probability that this interaction is a true positive.

In recent years, various graph-based clustering algorithms have been developed for detecting protein complexes and functional modules in protein interaction networks. According to whether the algorithm can identify overlapping clusters, these algorithms can be classified into two types: Non-overlapping clusters detecting algorithms and overlapping clustering identifying algorithms. These algorithms can also be divided into the follows: density-based and local search algorithms, hierarchical clustering algorithms, and other optimization-based algorithms, according to different definition and ideas.

### Density-based and local search algorithms

Based on the assumption that the members in the same protein complex and functional module strongly bind each other, a cluster can be referred as a densely connected subgraph within a protein interaction network. Several algorithms for finding dense subgraphs have been proposed.

The density (*d*) of a subgraph with *n* vertices and *m* edges is generally defined as *d=*2*m*/(*n*(*n-*1)) [11]. A dense subgraph is a clique when its density equals to 1, that is, every two vertices in which are connected by an edge. Spirin and Mirny [11] detected protein complexes and functional modules by enumerating all the maximal cliques. In general, the enumeration of all cliques within a graph is a NP-complete problem. Fortunately, protein interaction networks are scale-free and very sparse. Thus, this could be done quickly. However, only mining maximal cliques can not accurately represent the real structures of protein complexes and functional modules. This is because that the protein-protein interactions available are not complete and the protein interaction networks have the false negatives.

To avoid this limitation, Spirin and Mirny [11] introduced two new approaches: superparamagnetic clustering (SPC) and Monte Carlo optimization (MC). SPC uses an analogy to the physical properties of an inhomogenous ferromagnetic model to find highly-connected clusters in a large graph. MC formulates the problem of finding highly connected clusters as an optimization problem: find a set of *n* vertices that maximizes the function *d*. It starts with a connected set of *n* vertices randomly picked on the graph and proceeds by “moving” selected nodes along the edges of the graph to maximize *d*. Moves are accepted according to Metropolis criteria. In [11], the comparison of MC and SPC algorithms have been done, and the comparison results show a better performance of MC for clusters that share common vertices and for high density graphs, whereas SPC has an advantage identifying clusters that have very few connections to the rest of the graph.

Bu *et al.*[12] proposed a quasi-clique algorithm to find clusters. In their studies, they used the spectral analysis method to protein interaction networks and represented the network as a bi-directed graph which was denoted by a symmetric n*n adjacent matrix. Their key idea is that the proteins corresponding to absolutely larger components tend to form a quasi-clique for each eigenvector with a positive eigenvalue. To quantify a quasi-clique's tendency to form a cluster, Bu *et al* also used the density (in [12], they call it clustering coefficient, however, the two definitions are the same for a subgraph with *n* vertices and *m* edges, ie. 2*m*/(*n*(*n*-1)).). Except quasi-cliques, Bu *et al* also detected the quasi-bipartites as clusters. Cui *et al.*[13] also developed an efficient algorithm for finding cliques and near-cliques in protein interaction networks and showed a quasi-clique as well as a clique often represented a biologically meaningful unit such as functional module or protein complex.

More recently, Xiong *et al*[14] applied an association pattern discovery method to find the ‘hypercliques’ in the yeast protein interaction network. A hyperclique pattern is defined as a type of association pattern containing proteins that are highly affiliated with each other. Their studies revealed that proteins within the same hyperclique pattern tend to present in the protein complex together, also more likely perform the same function and participate in the same biological process. The most important contribution of their studies is that they discussed the identified hypercliques with 3-D structures, which has hardly been done in other papers’ validation of clusters. Their 3-D structural views show that proteins within a hyperclique pattern physically interact with each other.

In addition to the above mentioned methods related to cliques, another effective approach for clustering protein interaction networks is molecular complex detection algorithm (MCODE), which is proposed by Bader and Hogue [15]. MCODE consists of three stages: vertex weighting, complex prediction and optionally post-processing. In the first stage, MCODE weights all the vertices based on the core clustering coefficient. Different from the standard clustering coefficient, the core clustering coefficient of a vertex *v* is defined to be the density of the highest *k*-core of the immediate neighborhood of *v* (vertices connected directly to *v*) including *v*. A *k*-core is a graph of minimal degree *k.* Once the weights are computed, MCODE seeds a cluster with the highest weighted vertex and recursively moves outward from the seed vertex. A new vertex will be added to the cluster if its weight is larger than a given threshold. By such a greedy fashion, MCODE can isolate densely connected regions iteratively. In the post-processing step, MCODE filters or adds proteins based on connectivity criteria. MCODE has been a Cytoscape [17] plugin for detecting clusters in a network and used in several recent publications [18,19]. Zhang *et al*[18] created a protein-protein relationship network (PPRN) by using a kernel-based integration of protein interaction data and protein functional annotation data. They applied MCODE to the created PPRN network and the original protein interaction network, respectively. Their experiment results showed that the functional annotation could improve the ability of prediction of complexes. More recently, Cline *et al*[19] integrated biological network and gene expression data and identified putative complexes and functional modules by using MCODE. However, MCODE cannot guarantee that the predicted clusters are highly connected to each other, since the highly weighted vertices may not be highly connected to each other. Moreover, many proteins are left ungrouped into any cluster by MCODE in practice [20].

**Figure 1:**
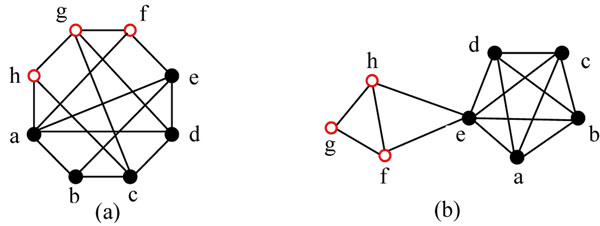
**Two typical graphs of the same size and density**[20]

The aim of the previous density-based algorithms is to detect the densely connected subgraphs. However, ensuring density alone is not enough for this aim, just as discussed in [21]. Altaf-UI-Amin *et al.*[21] illustrated this question by exampling two typical graphs of the same size and density (both consist of 8 vertices and are of density 0.5), as shown in Fig. 1.

From Fig.1 we can see that the topologies of these two graphs Fig.1 (a) and Fig.1 (b) are very different, though they have the same size and density. Fig.1 (a) looks more likely to be a single cluster than Fig.1 (b).

To mine dense subgraphs, Altaf-UI-Amin *et al*[21] proposed a new concept “periphery” and developed an algorithm DPClus based on the combination of density and periphery. For a given cluster *k* with density *d_k_*, the cluster property *cp_vk_* of any vertex *v* is defined as *cp_vk_=*|*E_vk_|*/(*n×d_k_*)*,* where |*E_vk_|* is the total number of edges between the vertex *v* and the vertices of cluster *k* and *n* is the number of vertices in cluster *k.* Similar to MCODE, DPClus also weightes all the vertices in its first step and started at a highest weighted vertex. In DPClus, a vertex’s weight is defined as the sum of the weights of the edges connected to the vertex and the weight of an edge (*u,v*) is the number of the common neighbors of the vertices *u* and *v*. DPClus takes the highest weighted vertex as an initial cluster and extends the cluster gradually by adding vertices from its neighbors. All neighbors are sorted by their priorities. A neighbor’s priority to a cluster is determined by the sum of the weights and the number of the edges between the neighbor and the vertices in the cluster. DPClus uses two parameters *d_in_* (a value of minimum density) and *cp_in_* (a minimum value for cluster property), to determine whether a neighbor should be added to the cluster. Once a cluster is generated, DPClus removes it from the graph. Then, the weights of all the vertices in the remaining graph are recomputed and the next cluster is formed in the remaining graph. The process goes on until no edge is left in the remaining graph. In such cases, DPClus can only generate non-overlapping clusters. To generate overlapping clusters, DPClus extends the non-overlapping clusters by adding their neighbors in the original graph (rather than in the remaining graph). The contribution of DPClus is that the concept “periphery” is proposed to distinguish different graph topologies from the same densities. However, its drawback is that a new cluster is removed from the graph and the vertex weights are needed to be recomputed based on the remaining graph. Such operations are not only time consuming, but also may neglect some useful biological information.

More recently, Li *et al*[22] investigated the structures of known protein complexes in MIPS and revealed that most protein complexes have a very small diameter and a very small average vertex distance. Li *et al*[22] proposed an algorithm IPCA for clustering protein interaction networks based on the combination of vertex distance and subgraph density. Similar to DPClus, IPCA also consists of four stages: weighting vertex, selecting seed, extending cluster, and extend-judgment. However, the rules of IPCA and DPClus for expanding clusters and weighting vertices are different. Especially, they look for different topological structure for the identified clusters. IPCA uses diameter (or average vertex distance) and interaction probability *IN_vk_* to determine whether a neighbor *v* should be added to a cluster *k*. For a cluster *k*, the interaction probability *IN_vk_* of a vertex *v* to it is defined as *IN_vk_=|E_vk_|*/*n.* In [22], Li *et al* discussed the relationships among *IN_vk_, cp_vk_*, and *d_k_*. One of the attractive features of IPCA is that, unlike DPClus, it will generate overlapping clusters directly and does not need to consider the identified clusters’ neighbors in the original graph. Moreover, IPCA avoids the recomputation of vertex weights, which is time consuming.

### Hierarchical clustering algorithms

Hierarchical clustering is one of the most common methods of classification used in biology and bioinformatics. In recent years, hierarchical clustering algorithms have been used widely for the analysis of biological networks. The hierarchical organization of biological networks has been frequently discovered. For example, Yook *et al.*[23] discovered the underlying hierarchical structure in the yeast protein interaction network, and Ng *et al.*[24] extended the studies from one species (*S. cerevisiae*) to seven species (*E*. *coli, H. pylori, C. elegans, D. melanogaster, H. sapiens, M. musculus, and S. cerevisiae*), and Farkas *et al.*[25] found out the hierarchical organization of the yeast transcriptional regulatory network. Generally, the hierarchical clustering algorithms can represent the hierarchy of a complex network as a tree. According to the difference of the processes of the tree’s construction, hierarchical clustering algorithms can be divided into two classes: the agglomerative algorithm and the divisive algorithm. Agglomerative algorithms start at the top of the tree and iteratively merge vertices, whereas divisive algorithms begin at the bottom and recursively divide a graph into two or more subgraphs. For merging vertices or separating the graph, various heuristic rules have been used, such as betweenness centrality [26-36], clustering coefficient [29,37-41], minimum cut [42], and etc.

### Betweenness centrality-based clustering algorithms

Betweenness centrality is an important metric for analyzing protein interaction network [26]. There are two types of betweenness centrality: the vertex betweenness and the edge betweenness. The vertex betweenness centrality *BC*(*v*) of a vertex *v*∈*V* is the sum over all pairs of vertices *s,t*∈*V,* of the fraction of shortest paths between *s* and *t* that pass through *v*, as the formula (1) [26]:(1)

where σ*_st_*(*v*) denotes the total number of shortest paths between *s* and *t* that pass through vertex *v* and σ*_st_* denotes the total number of shortest paths between *s* and *t*. Similarly, the edge betweenness centrality *BC*(*e*) of an edge *e* ∈ *E* is defined as formula (2) [27,28]:(2)

where σ*_st_*(e) denotes the total number of shortest paths between *s* and *t* that pass through edge *e.*

As suggested by Girvan and Newman [27], the edges with highest betweenness values are least central, which are most likely to lie between clusters, rather than inside a cluster. Thus, one can separate a network into clusters by removing edges from the original graphs based on the edge betweenness centrality. Girvan and Newman [27] developed a divisive algorithm (G-N algorithm) to detect community structures in complex networks as follows:

(1) Calculate the betweenness for all edges in the network;

(2) Remove the edge with the highest betweenness;

(3) Recalculate betweennesses for all edges affected by the removal;

(4) Repeat from step (2) until no edges remain.

The output of algorithm G-N is a tree (or dendrogram) which represents an entire nested hierarchy of possible community divisions for the network. However, one can not know where the tree should be cut to get a good division for the given network. In general, we would like to get the best division. To obtain this aim, Newman and Girvan proposed a measure, called *modularity*[28], to evaluate the quality of a particular division of a network. Let the network be divided into *k* clusters and element *e_ij_* of matrix *e* (a *k×k* symmetric matrix corresponding to the *k* clusters) be the fraction of all edges in the network that link vertices in cluster *i* to vertices in cluster *j*. Then, a modularity measure *Q*[28] is defined as , where *e_ij_* represents the fraction of edges that connect to vertices in cluster *i* and  gives the fraction of edges that connect vertices in the same cluster. A larger value of *Q* indicates that the division is better. Typically, the values of *Q* fall in the range from about 0.3 to 07 [28].

In stead of modularity measure, another effective method for obtaining good division is to define module quantitatively. There are several definitions of modules which have been proposed [29-31,39,40], as shown in Table 1.

**Table 1:** **Different Definitions of module in protein interaction network**[29-31,39,40]

Module Definitions			References

Module Names	Computational Formula	Descriptions
Strong Module		In a strong module each vertex has more connections within the module than with the rest of the graph.	[29]

Weak Module		In a weak module the sum of all degrees within subgraph *H* is larger than the sum of all degrees toward the rest of the network.	[29]

Chen *et al.*		A combination of weak module and a new less stringent condition, which is that, collectively, the in-degrees of the vertices in the subgraph are significantly greater than the out-degrees.	[30]

Luo *et al.*		A subgraph *H ⊂ G* is a module if its modularity *MH* >1. In the definition, *ind*(*H*) denotes the number of edges within *H* and *outd*(*H*) denotes the number of edges that connect *H* to the remaining part of *G.*	[31]

*λ*-module		λ-module is a general version of weak module. When *λ*=1, it would be the same as weak module defined by Radicchi *et al.* By changing the values of parameter λ*,* one can get different modules in the protein interaction networks.	[39]

λ*-module		λ*-module is a more general version of *λ*-module, which is used for weighted protein interaction networks.	[40]

In Table 1, different criterions are shown that the given subgraph *H* ⊂ *G* is a module.

denotes the “in-degree” of vertex *i* (i.e. the number of edges connecting vertex *i* to other vertices belonging to *H*) and  denotes the “out-degree” of vertex *i* (ie. the number of edges connecting vertex *i* and other vertices in the rest of the graph *G*). Let *k_i_* be the degree of vertex *i*. Then, .  and are the weighted “in-degree” and “out-degree” of vertex *i,* respectively.

Based on the division process of algorithm G-N, Radicchi *et al*[29] proposed two types of module definitions: strong module and weak module. They gave a new self-contained algorithm to identify modules from networks as follows [29]:

(1) Choose a definition of module (*strong module* or *weak module*);

(2) Compute the edge betweenness for all edges and remove those with the highest score.

(3) If the removal does not split the (sub-)graph go to point 2.

(4) If the removal splits the (sub-)graph, test if at least two of the resulting subgraphs fulfill the definition. If they do, draw the corresponding part of the dendrogram.

(5) Iterate the procedure (going back to point 2) for all the subgraphs until no edges are left in the network.

Later, Chen *et al*[30] extended the G-N algorithm for clustering in weighted protein interaction network. They suggested that the shortest path should be computed based on edge weights since the protein interactions are not all equally important. They weighted the edges by using microarray datasets. They combined the weak module and a new less stringent condition, which was that, collectively, the in-degrees of the vertices in the subgraph were significantly greater than the out-degrees, to identify the modules in the protein interaction networks. Another contribution of their work is that they modified the original definition of edge betweenness to try to eliminate the unbalanced partition in it. The modified betweenness of an edge is the maximum number of *non-redundant* all-against-all shortest paths passing through it, i.e., the end points must be distinct when the number of shortest paths for an edge is counted [30].

More recently, Luo *et al*[31] modified the definition of weak module by extending the concept of degree from single vertex to subgraph. They suggested that the edges inside a subgraph should not be counted multiple times (in the weak module definition, each edges are counted two times). In their definition, the “*in-degree*” of a subgraph *H* ïƒŒ *G* was defined as the number of edges within *H* and the “*out-degree*” of *H* was defined as the number of edges that connect *H* to the remaining part of *G*. In fact, the “*in-degree*” of *H* is just half of the sum of degrees of vertices within *H,* as shown in Table 1*.* Thus, the module definition of Luo *et al* is more stringent than weak module. Based on the new definition of module and G-N algorithm, Luo *et al*[31] developed an agglomerative algorithm MoNet. MoNet initialed each vertex as a cluster and then assembled the clusters into modules by gradually adding edges to the clusters in the reverse order of deletion by the G-N algorithm. In [31], Luo *et al* compared the MoNet modules, the weak modules and the strong modules defined by Radicchi *et al*[29]. The comparison results showed that MoNet modules represented stronger coclustering of related genes and were more robust to ties in betweenness values.

The betweenness-based clustering algorithm has been used widely due to its good performance in hierarchical clustering. It has also been used to predict biological function in protein interaction networks [32] and predict missing links in complex networks [33]. However, most of the betweenness-based clustering algorithms grouped vertices into separated clusters. To allow vertices to be presented in multi-modules, Pinney *et al*[34] proposed an alternative formulation of betweenness-based decomposition, which was based on vertex betweenness instead of edge betweenness. They guaranteed to detect overlapping modules by dividing the network at the vertices with the highest betweenness and copying such vertices into the divided subnetworks.

Another drawback of betweenness-based clustering approaches is that it is computationally expensive because it requires the repeated evaluation for each edge in the system [28,29]. Up to now, the best algorithm of calculating betweenness for all *m* edges in a graph of *n* vertices is in time O(*mn*) [29]. Thus, the complexity of repeated calculation of each edge betweenness is O(*m*^2^*n*). As pointed out by Radicchi *et al*[29], the betweenness-based approaches are unfeasible to be used in networks larger than 10000 vertices. To reduce the running time, one might be tempted to calculate the betweennesses of all edges only once and removing the edges with the largest betweenness orderly. Girvan and Newman [28] discussed this strategy and found that it did not work well because there was no guarantee that all edges between modules would have high betweenness when there were more than one edges between two modules. Another appealing solution for improving computational efficiency is parallelization. Yang *et al*[35,36] developed a parallel edge-betweenness clustering tool for implementation of Girvan and Newman's clustering algorithm that achieved almost linear speed-up for up to 32 processors.

### Clustering coefficient-based clustering algorithms

Clustering coefficient is first proposed to describe the local property of vertex and used widely to analyze the topologies of protein interaction networks [16,37,38]. To develop fast hierarchical clustering algorithm, Radicchi *et al*[29] began to consider using the local quantity instead of the global quantity (betweenness centrality) to single out the edges connecting different clusters. They generalized the clustering coefficient of a vertex to an edge and defined it as the number of triangles to which a given edge belonged, divided by the number of triangles that might potentially include it. Given an edge *e*(*u*,*v*), its clustering coefficient [29] is defined as:(3)

where  is the number of triangles built on that edge e(*u,v*) and min [(*k_i_*-1),( *k_j_*-1)] is the maximal possible number of them. The idea behind the use of this definition in [29] is that many triangles exist within clusters and those edges between different clusters are included in few or no triangles. Thus, edges with small values of  tend to lie between different clusters. Based on this idea, Radicchi *et al*[29] developed a fast divisive algorithm using the same steps as their proposed self-contained algorithm. In their algorithm, they also extended the definition from triangles to higher order cycles, such as squares, and defined the clustering coefficient of order *g* as [29] where  is the number of cyclic structures of order *g* built on the edge *e*(*u*,*v*) and  is the number of possible cyclic structures of order *g.*

However, this definition is not feasible when the network has few triangles or higher order cycles. To avoid of the limitation, Li *et al*[39] redefined the edge clustering coefficients again by calculating the common neighbors instead of triangles, as shown in formula (4):(4)

where *N_u_* is the set of neighbors of vertex *u* and *N_v_* is the set of neighbors of vertex *v*, respectively.

Based on the definitions of edge clustering coefficients and *λ*-module (as shown in Table 1), Li *et al*[39] proposed a fast agglomerative algorithm FAG-EC. FAG-EC can generate different size of clusters by changing the value of parameter *λ*. More recently, Li *et al*[40] gave a new definition of edge clustering coefficient in weighted protein interaction networks, as shown in formula (5):

(5)(5)

where *w*(*u*,*v*) denotes the weight of edge *e*(*u*,*v*), *I_u,v_* denotes the set of common vertices in *N_u_* and *N_v_* (i.e. *I_u,v_* = *N_u_*∩*N_v_*). Correspondingly, Li *et al* defined λ*-module of weighted protein interaction networks, as shown in Table 1. The experimental results in [40] shows that the new definition of edge clustering coefficient and *λ* *-module of weighted protein interaction networks can help improve the accuracy of clustering. Another contribution of their work is that FAG-EC and HC-Wpin can identify the functional modules in a hierarchy by changing the values of parameter *λ* and such hierarchical organization of modules approximately corresponds to the hierarchical structure of GO annotations. More attractive strength of FAG-EC and HC-Wpin is their efficiencies. The total time complexities of FAG-EC and HC-Wpin are both O(*k^2^m*). As is well known the scale-free of protein interaction networks, *k* is very small and can be considered as a constant. Thus, FAG-EC and HC-Wpin are very fast which can be used in large protein interaction networks as the protein-protein interactions accumulate.

Recently, Wang *et al*[41] combined the local metric (Clustering Coefficient, which is named Commonality in [41]) and the global metric (Betweenness) to generate clusters for balance and consistency.

### Other hierarchical clustering algorithms

Besides the two typical metrics discussed above, a number of other metrics have also been suggested to be used in the hierarchical clustering algorithms. Hartuv and Shamir [42] used the minimum cut to remove edges recursively and developed a divisive algorithm HCS for the discovery of highly connected subgraphs. Recently, HCS [43] has been successfully applied in clustering the protein interaction networks. Arnau *et al.*[44] developed a hierarchical clustering algorithm, named UVCLUSTER, based on the shorted path between any two vertices on protein interaction networks. Lu *et al.*[45] suggested a simple graphical measure to depict the relationship between proteins and extracted the topological information of the network, such as quasi-cliques and spoke-like modules, into a clustering tree. Several similarity measures, such as diffusion kernel similarity, shortest path based similarity, and adjacency matrix based similarity, are evaluated by Wang *et al.* in [46]. They proposed a nonnegative matrix factorization (NMF)-based method with the usage of diffusion kernel similarity for clustering complex networks and biological networks.

The definition of similarity metric or distance measure is a crucial step for hierarchical clustering. How to evaluate the metrics is another challenge in hierarchical clustering. Two evaluation schemes suggested by Lu et al, which are based on the depth of hierarchical tree and width of ordered adjacency matrix, may be useful. Moreover, Chen et al [47] gave a formal definition of similarity metric and discussed the relationship between similarity metric and distance metric, they also presented general solutions to normalizing a given similarity metric or distance metric, which have provided a theory basis for constructing metrics.

The obvious advantage of hierarchical clustering approach is that it can present the hierarchical organization of protein interaction networks. Its drawback is that it can not generate overlapping clusters except that special pre-processing or other strategies are used. In addition, the hierarchical clustering approaches are known to be sensitive to the noisy data in protein interaction networks [48].

### Other optimization-based algorithms

In addition to the density-based and local search algorithms and hierarchical clustering algorithms, some other optimization-based algorithms are also frequently used. For example, King *et al*[49] proposed the Restricted Neighborhood Search Clustering (RNSC) algorithm which aimed at exploring the best partition of a network by using a cost function. RNSC starts with randomly partitioning a network, and iteratively moves a vertex from one cluster to another to decrease the total cost of clusters. It ends up when some moves have been reached without decreasing the cost function. It can get the best partition by running multi-times. Its drawback is that it needs the number of clusters as prior knowledge and its results depend heavily on the quality of initial clustering.

Another optimization model for the discovery of clusters was proposed by Newman and Girvan [28], in which a quantitative measure, called modularity Q, was used to evaluate the quality of a partition for a given network. The detection of clusters in a network thus was translated into searching for the divisions of a network with high modularity Q. This optimization model has been widely adopted, and several algorithms have been developed to optimize modularity Q. For example, Guimera and Amaral[50] and later Danon *et al*.[51] suggested to optimize Q by using simulated annealing. Unfortunately, optimizing Q is NP-hard [52], and optimization by simulated annealing requires too much computational effort and is not suitable for large networks. Therefore, a number of alternative heuristic methods have been developed, such as greedy algorithms [53], extremal optimization [54], and spectral approach [55-57].

Recently, Hwang *et al.*[58] presented a novel functional module detection algorithm STM by using a pharmaco dynamic signal transduction network model.

STM consists of four steps [58]:

(1) Compute signals transduced between all vertex pairs;

(2) Select cluster representatives for each vertex;

(3) Formation of preliminary clusters;

(4) Merge preliminary clusters.

In STM, the Erlang distribution is used to model the signal transduction behavior of the network. STM considers only the least resistance paths between protein pairs in a network and propagates the occurrence probability through a shortest path between a protein pair. More recently, Hwang *et al* extended STM to CASCADE [59], in which the occurrence probability of a series of pairwise interactions is propagated through the protein interaction network via the QAP (Quasi all paths) extension. The QAP algorithm enumerates all the possible paths approximately.

Among others, the Markov Cluster Algorithm (MCL) [60,61] has been proved to be a very successful clustering procedure, which has been developed in different languages, such as C, R, JAVA and PERL. MCL simulates random walks on networks, by alternating two operations: expansion and inflation. It constructs a stochastic “Markov” matrix representing the transition probabilities between all pairs of vertices. As MCL is fast and scalable, it has been used for predicting protein family [61] and in a number of other domains. Pereira-Leal *et al*[62] transformed the protein interaction network into a line graph and then applied MCL to find functional modules. The line graph is reconstructed from the original graph by using vertices representing edges and edges representing shared vertices. The advantages of line graph being used is that it is more highly structured than the original graph by taking into account the higher-order local neighborhood of interactions. In a recent comparison of graph clustering algorithms [10], MCL was shown to be the most robust algorithm for identifying protein complexes and outperforming SPC [11] and RNSC [48]. More recently, another comparison work by Vlasblom J and Wodak [63] showed that MCL outperformed the Affinity Propagation (AP) for the partitioning of protein interaction graphs. Cannataro *et al*[64] have provided a web portal, allowing remote users to access MCL functions through the Internet, for the identification of protein complexes.

Furthermore, in the recent past, some novel optimal clustering approaches have been proposed for the discovery of protein complexes or functional modules. Mete *et al.*[65], for example, proposed a new structural clustering algorithm, called SCAN, for detecting functional modules from large biological networks. The basic idea behind SCAN is that two vertices should be assigned into a cluster or not according to how they share neighbors. In other words, SCAN is a method based on common neighbors. Both connectivity and local structures are used in SCAN. One contribution of SCAN is that it not only can achieve an optimal clustering of the protein interaction network, but also can identify hubs and outliers. Luo *et al*[66] investigated the core and periphery structures in protein interaction networks. The model of core/periphery structure was first formalized by Borgatti and Everett [67] in social networks. In the core/periphery structure model, members in the core set are cohesively connected to each other, and those in the periphery set are loosely connected to the core members.

In [66], the core was defined as a local maximal *k*-plex [68] with *k*≤*n*/2, for a given *k*, where *n* was the number of vertices in the cluster, and the peripheries of a core was defined as the set of vertices that were not in the core and whose distances to any member in the core were equal to *l* (only 1- and 2-peripheries were mainly considered in [66]).

### Finding overlapping clusters

In recent years, much attention has been focused on the clustering algorithms for finding overlapping clusters. For the overlapping clusters, each protein may be involved in multiple complexes or functional modules. This is particularly true of protein interaction networks for most proteins having more than one biological function. Some of the above mentioned clustering algorithms, such as STM [48], can be used for generating overlapping clusters. In this subsection, we mainly discuss the algorithms which are proposed for the purpose of finding overlapping clusters.

In 2005, Palla *et al.*[69] investigated the overlapping structures in complex networks and proposed a Clique Percolation Method (CPM). CPM generates overlapping clusters by finding *k*-clique percolation communities. A *k*-clique is a complete subgraph of size *k.* Two *k*-cliques are said to be adjacent, if they share exactly *k*-1 vertices. A cluster is defined as a union of all *k*-cliques that can be reached from each other through a series of adjacent *k*-cliques. Based on CPM, a powerful tool CFinder for finding overlapping clusters has been developed by Adamcsek *et al.*[70]. Though with many attractive characters, CPM is limited in the followings: 1) its results are highly correlated to the value of parameter *k;* 2) the proteins not included in any *k*-cliques are neglected. To overcome the disadvantages of CPM, people often adopt some pre-processing or post-processing when using it. Jonsson *et al.*[71] constructed a weighted protein interaction network for rat proteome and used CPM to identify key protein clusters involved in cancer metastasis. Zhang *et al* proposed two types of strategies: size control [72] and line graph transformation [73] when using CPM. For size control, they used *k*=3 to generate initial clusters and then iteratively used *k*+1 to separate the clusters of size larger than a given integer *S* until all the identified clusters of size were less than *S*.

Zhang *et al*[74] suggested a simple method, called MC(2), to identify functional modules by enumerating and merging cliques and applied it to a yeast protein interaction network. Instead of finding all the maximal cliques, Li *et al*[75] proposed to detect the local cliques for each protein and then to merge the detected local cliques according to their affinity. The affinity between two identified clusters is determined by their intersection sets and each cluster’s size. Two clusters are more similar and have larger affinity if they have larger intersection sets and similar sizes. For best matching with the known complexes, the value of affinity is suggested to be 0.4. Considering the incompleteness of current protein interaction data and the fact that many dense but non-clique subgraphs for each vertex could also form parts of a complex, Li *et al*[76] proposed an improved algorithm DECAFF based on LCMA. In DECAFF, They used a Hub removal algorithm to detect multiple dense subgraphs with densities larger than the given threshold δ.

Another method based on clique for identifying overlapping clusters is COD (Complex Overlap Decomposition) proposed by Zotenko *et al.*[77]. COD requires the network satisfying certain mathematical properties. It builds on chordal graph, which does not contain chordless cycles of length greater than three. Thus, the first step of COD is to construct a chordal graph from the original graph by graph modification. Each chordal graph has a corresponding clique tree representation or clique tree [78]. The vertices in the tree are maximal cliques. The topology of the tree is determined by the structure of overlaps between the maximal cliques. The drawback of COD is that it will not work if the modified graph is not chordal.

The essential proteins have always been counted as having a close connection to the overlapping clusters [79-82].Typically, a few highly connected vertices, also known as hubs, tend to be essential proteins [4]. These hubs generally are linked to several protein complexes or functional modules. Ucar *et al.*[79] proposed a refinement method based on neighborhoods and the biological importance of hubs. They detected the overlapping clusters by using hub duplication. Li *et al.*[80] suggested a graph split and reduction method to discover overlapping clusters with the restriction that only the highly connected hubs could belong to more than one functional modules. Pei *et al.*[81] developed a seed-refine algorithm for detecting the overlapping clusters by using a two-layer seeding heuristic method to find good seeds and adopting a subgraph refinement approach for controlling the overlap between clusters. The information flow-based approach for identifying overlapping clusters proposed by Cho *et al.*[48,82] was also based on the informative proteins selection. In [82], the informative vertices were selected based on the weighted connectivity where the weight was estimated by using coexpression profiles of normalized microarray gene expression data from SMD [83]. Later in [48], Cho *et al.* combined the flow-based approach with two new metrics: semantic similarity and semantic interactivity, where Gene Ontology (GO) annotations were used to weight protein-protein interactions. Different methods adopted for the selection of essential proteins will result in different overlapping clusters. Thus, to select the informative vertices more exactly will help to identify the overlapping clusters more accurately.

Moreover, some extended hierarchical clustering algorithms can also be used for the identification of overlapping clusters. Pinney *et al*[34], for instance, proposed an alternative formulation of betweenness-based decomposition, which was based on vertex betweenness instead of edge betweenness. They guaranteed to detect overlapping modules by dividing the network at the vertices with the highest betweenness and copying such vertices into the divided subnetworks. Similarly, Gregory developed an algorithm CONGA [84] based on the key definition of “split betweenness” to decide when to split vertices, which vertices to split, and how to split them.

In addition, the algorithms of detecting overlapping community structures in other complex networks, such as fuzzy clustering [85], EAGLE [86], and node fitness-based clustering [87], probably can also be used in protein interaction networks.

## Combination with other information and ensemble

### Integration of Multiple Sources

The above discussed methods for identifying clusters are mostly based on graph theoretic properties solely and only require the protein-protein interaction data. Unfortunately, protein interaction networks, as we all know, can not avoid of the false positives and false negatives [10]. To lessen the effect of them, one can add a pre-processing [88] for evaluating the reliability of the interactions, filtering the false positives, or predicting the false negatives, to improve the robustness of the clustering algorithms. Other than the adoption of pre-processing, several authors have suggested to develop robust clustering algorithms by integrating data from multiple sources, such as genomic data [89-91], structure information [92], gene expression [19,93-101], Gene Ontology (GO) annotations [48,102,103], etc. The approaches differ in the way the sources are combined.

Jiang and Keating [89] described the first integrative framework, named AVID which integrates experimental results with sequence information, for the discovery of functional relationships among proteins. Zheng *et al.*[90] integrated seven genomic features and four experimental interaction data sets by using a Bayesian-networks-based data integration approach. From the inferred protein interaction networks, they implemented algorithm MCL to detect protein complexes. Zhang *et al.*[91] developed another multi-step but easy-to-follow framework for the detection of protein complexes which estimated the affinity between each pair of proteins based on their co-purification patterns derived from MS data. Dittrich *et al.*[92] presented an integrated exact approach for clustering protein interaction networks based on integer-linear programming and its connection to the prize-collecting Steiner tree problem. Their approach allows a smooth integration of data from various sources. Instead of yeast, they applied their method on a large interaction network of HPRD in combination with associated survival data.

Jung *et al.*[93] presented a method to detect protein complexes based on the integration of protein-protein interaction data and mutually exclusive interaction information which were drawn from structural interface data of protein domains. PSIMAP [105], a tool and Database for constructing interactomes, provides interfacial residue pairs in physical domain-domain interactions. By excluding interaction conflicts, Jung *et al.*[93] extracted cooperative sets of proteins as the Simultaneous Protein Interaction Cluster (SPIC) from the protein interaction network. Then, they applied conventional graph-based clustering algorithms, MCODE [14] and LCMA [75], to estimate the density of clusters.

Owing to the attribute that members in a cluster typically perform a specific biological function [106], several clustering algorithms have been proposed with a combination of protein-protein interaction data and gene expression data. For example, Jansen *et al.*[94] related whole-genome expression data with protein-protein interactions and scored expression activity in complexes. Hanisch *et al.*[95] proposed a Co-clustering methodology by using a distance function which combined similarity of gene expression profiles with network topology. Ideker *et al.*[96] developed a clustering algorithm for the discovery of active subnetworks which showed significant changes in expression over a particular subset of the conditions. Unfortunately, this method requires an activity p-value for every measurement, a situation which is rather uncommon [97]. Segal *et al.*[98] introduced a probabilistic graphical model to detect functional modules from gene expression measurements combined with protein-protein interaction data, in which a module was expected to contain a significant portion of the possible interactions. Maraziotis *et al.*[99] presented an algorithm to identify dense subnetworks in the weighted graph by expanding a kernel protein sets from a seed protein via integration of protein interaction and gene expression data. The weighted graph was constructed by using the gene expression information. Cho *et al.*[100] also introduced an algorithm based on informative protein selection from a weighted graph where the weight was computed by using co-expressional profiles. Moreover, graph reduction and hierarchical clustering based on minimum cut were also used in [100]. Recently, Lu *et al.*[101] proposed a hierarchical clustering algorithm based on the integration of high-throughput protein-protein interaction data with the added subcellular localization and expression profile data. They were the smart few who distinguished protein complexes from functional modules when clustering in protein interaction networks.

More recently, Ulitsky and Shamir [97] transformed the high-throughput data into similarity values, on the basis of which they found clusters, named as Jointly Active Connected Subnetworks (JACSs), which manifested high similarity. Also, a program called MATISSE (Module Analysis via Topology of Interactions and Similarity SEts) was developed for the discovery of JACSs. The problem of seeking for JACSs was actually to discover the subnetworks of maximum likelihood by transforming edge weights to attain probabilistic meaning. For the problem of discovering the heaviest-subnetwork is computationally hard, Ulitsky and Shamir introduced several heuristic methods, see in [97]. One advantage of MATISSE is its flexibility. Except gene expression similarity, other similarity measures, such as functional similarity or similarity in protein-DNA binding profiles, can also be used in MATISSE. Even more recently, Ulitsky and Shamir [102] presented another novel confidence-based method for extracting functionally coherent co-expressed gene sets, named Co-Expression Zone ANalysis using NEtworks (CEZANNE), by using expression profiles and confidence-scored protein interactions. CEZANNE is available as part of the MATISSE software.

Except for gene expression data, authors also usually combined protein interaction networks with GO annotations. Typically, the flow-based approach proposed by Cho *et al.*[48], as already discussed, is a method combined with GO annotations. Besides, Lubovac *et al.*[103] suggested a Semantic WEights for MODule Elucidation (SWEMODE) by using an alternative measure, called weighted clustering coefficient, and a weighting scheme according to semantic similarity between the proteins. Turanalp and Can [104] mapped known functional annotations onto a protein interaction network and adopted a frequent pattern identification technique, PPISpan, to detect recurring functional interaction patterns instead of single clusters.

With the rapidly expanding resource of microarray data and other biological information, such as structure profiles [92] and phylogenetic profiles [107], combination with these information is believed to be an intriguing method to solve the problem of unreliable interaction data when clustering in protein interaction networks.

### Ensemble clustering framework

**Figure 2:**
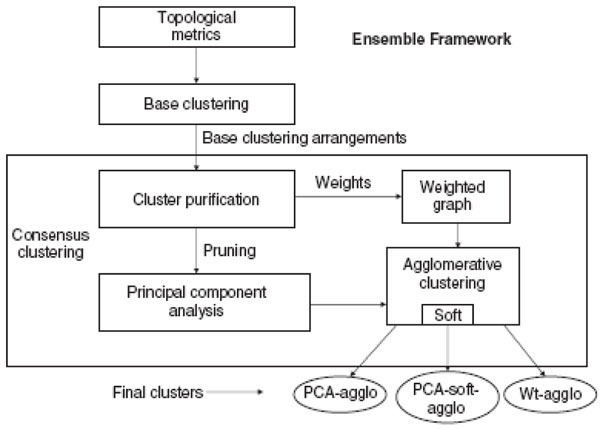
**Overview of the ensemble framework**[108]

Ensemble clustering [108,109] has been proposed to obtain a single, comprehensive consensus clustering by combining multiple, diverse and independent clustering results. As different datasets may be generated using different approaches and even from the repeated application of a given approach with different parameters when clustering in the same protein interaction network, ensemble clustering may be a good choice to get more desirable clustering results. Asur *et al.*[109] first presented an ensemble framework, as shown in Fig.2, for clustering in protein interaction networks.

In [109], initially three conventional graph partitioning algorithms: repeated bisections, direct *k*-way partitioning, and multilevel *k*-way partitioning, with two topology driven distance metrics were used to obtain six base clusterings, and then a consensus method based on Principal Component Analysis (PCA) was developed to reduce the dimensionality of the consensus problem. Asur *et al.*[109] also designed an adaptation to allow for soft ensemble clustering in protein interaction networks.

Another ensemble framework for clustering protein interaction networks was proposed by Greene *et al.*[110]. They first produced a collection of non-negative matrix factorizations (NMF) and then combined the factorizations to produce an improved clustering. NMF proposed by Lee and Seung [111] was adopted for accurately detecting overlapping groups. A latest study on clustering complex networks and biological networks by non-negative matrix factorization with various similarity measures can be seen in [112]. Consensus solution given by Greene *et al.*[110] was a soft hierarchical clustering.

As being in nascent stage, ensemble clustering approach inevitably faces some challenges for the discovery of protein complexes and functional modules. A series of crucial factors, such as choosing the basic clustering methods, building a consensus, and adapting for soft clustering, must be taken into account carefully.

## Validation and comparison of clustering methods

### Validation

Biological validation of the predicted clusters in protein interaction networks is very essential. As previous discussed, disparate results can be obtained from the same protein interaction network with different algorithms or even with the same algorithm where different parameters are chose. Therefore, different solutions must be carefully compared in order to select the approach and parameters which provide the best outcome. Validation is a process of evaluating the performance of the clustering or prediction results derived from different approaches. This section will introduce several basic validation approaches for clustering in protein interaction networks.

### ♦ Validation based on functional homogeneity

Previous studies have showed that proteins in the same cluster often have high functional homogeneity [49]. The functional homogeneity of proteins in a predicted cluster with known function annotation is generally evaluated with P-value, as shown in formula (6):

(6)(6)

where the predicted cluster *C* contains *k* proteins in the functional group *F,* and the entire protein interaction network contains |*V*| proteins. P-value with a hypergeometrical distribution shows the probability that a given set of proteins is enriched by a given functional group merely by chance. Smaller P-value indicates that the predicted cluster is not accumulated at random and is more significant biologically than one with a larger P-value. The function annotation can be obtained from MIPS [113] or GO (Gene Ontology) [114]. Different from MIPS, GO provides three types of annotations: molecular function, biological process, and cellular component which can all be used to assess the biological significance of each predicted cluster.

As the P-value of a single cluster is statistically not representative, a measure named clustering score, defined as formula (7), has been suggested to quantify the overall clusters.(7)

where *n_S_* and *n_I_* denotes the number of significant and insignificant clusters, respectively and *min*(*pi*) denotes the smallest P-value of the significant clusters *i (i=1 to n).* The *cutoff* is used to distinguish a significant cluster from insignificant clusters. We say a cluster is significant if its corresponding smallest P-value is lower than the *cutoff* value.

Another method for assessing the functional homogeneity of proteins within a predicted cluster is redundancy [62], as shown in formula (8):(8)

where *n* represents the number of classes in the classification scheme, and *p_s_*represents the relative frequency of the class in the predicted cluster. All values of *R* lie between 0 and 1. With this scoring system, clusters containing many proteins with highly consistent classifications will receive high scores (*R* closer to 1), whereas those with disparate or conflicting classifications will receive low scores (*R* closer to 0).

### ♦ Validation based on known complexes

To evaluate the performance of algorithms for clustering in protein interaction networks, a comparison of the predicted clusters (*Pc*) and the known complexes (*Kc*) is often done. The gold-standard data used as known complexes are available form those catalogued in the MIPS database [113]. The overlapping score *OS*(*Pc,Kc*) between a predicted cluster *Pc* and a known complex *Kc* is generally calculated by formula (9) [15,21,22]:(9)

where |*V_Pc_*∩*V_Kc_*| is the size of the intersection set of the predicted cluster *Pc* and the known complex *Kc*, |V*_Pc_*| is the size of *Pc* and |*V_Kc_*| is the size of *Kc*. A known complex and a predicted cluster are considered as a match if their overlapping score *OS*(*Pc,Kc*) is larger than a specific threshold δ*.* Generally, 0.2 is used in the literature [15].

Obviously, known complexes and predicted clusters are expected to be matched as many as possible. Sensitivity and specificity [15,22] are two important aspects to estimate how they are matched. Sensitivity is the fraction of the true-positive predictions out of all the true predictions, defined as *Sn=TP*/(*TP+FN*)*,* where *TP* (true positive) is the number of the predicted clusters matched by the known complexes with *OS*(*Pc,Kc*)*≥δ,* and *FN* (false negative) is the number of the known complexes that are not matched by the predicted clusters [15,22]. Specificity is the fraction of the true-positive predictions out of all the positive predictions, defined as *Sp=TP/*(*TP+FP*)*,* where *FP* (false positive) is equal to the total number of the predicted clusters minus *TP.* Generally, another integrated method, called *f*-measure, as shown in formula (10) [22], is also used to estimate the matching results by taking into account of both the sensitivity and the specificity.(10)

Also, we can determine a best matched known complex for a predicted cluster by minimizing the probability *P_ol_* of a random overlap between them. The *P_ol_* is defined as:(11)

where *i* is the number of the common proteins between the predicted cluster *Pc* and the known complex *Kc.* The smaller the *P_ol_* is, the more consistent they are.

One can also match the clustering result with the known protein complexes by building a contingency table *T*, as that has been done by Brohée and Helden [10]. Given *n* known complexes and *m* predicted clusters, the contingency table is a n*m matrix where row *i* corresponds to the *i^th^* known complex, and column *j* to the *j^th^* cluster. The value of a cell *T_ij_* indicates the number of common proteins that appear both in complex *i* and cluster *j.* In addition, some other measurements, such as positive predictive value (PPV), accuracy, and separation, can also be used to evaluate the match between a set of known complexes and a clustering result. More details about these measurements, the reader are referred to [10].

### ♦ Validation cased on other methods

Besides the above measurements, a comparison of the clustering results performed on protein interaction networks and on random networks is usually used. The random network requires having the same size and the same degree distribution as the original protein interaction network. Generally, one can get a corresponding random network by shuffling the edges between vertices in the original network [21,22]. Sometimes, a topology-based Modularity metric, as previous discussed, can also be used to estimate the performance of a clustering algorithm. It is mainly used to investigate whether the clustering algorithms group the highly connected vertices in a cluster. The proteins included in the same cluster, as reported in [115] by Zhang *et al.*, generally tend to share similar temporal expression profiles, subcellular localizations, and gene phenotypes, which support the functional relevance of modular organization. Moreover, the robustness of a clustering algorithm can be validated by different levels of graph alterations, such as proportions of edges added or deleted at random can be used to test the algorithm’s robustness against the false positives and false negatives.

### Comparison of clustering methods

Up to now, there have been few special works for quantitative evaluation of the clustering algorithms except for some comparison works that have been done in each proposed algorithm for demonstrating its validity. Only in 2006, a systematic quantitative evaluation of four clustering algorithms: MCL [60,61], MCODE [15], RNSC [49], and SPC [11] was done by Brohée and Helden [10]. They constructed a test graph using 220 known complexes represented as cliques and generated 41 altered graphs by randomly adding or removing edges in various proportions. Their comparison results show that MCL has the best performance on both simulated and real data sets and is robust to graph alternations. This comparison was done on unweighted networks, whereas the MCL and SPC algorithms can deal with weighted graphs and are likely to give better performances if weights are assigned to reflect the reliability of the interactions.

Tuji *et al.*[116] compared two different types of clustering algorithms: DPClus [21], a density based algorithm and G-N [28], a hierarchal clustering algorithm. Their comparison results show that each method has its own advantage. G-N algorithm may be better by taking into account the global structure of the network, but cannot eliminate ambiguities in its early step of clustering. By contrast, DPClus does not focus on any type of global optimization, but introduces local optimizing parameters which help for more precise detection. In the following Table 2, we give a rough comparison of 20 typical clustering algorithms for extracting clusters from protein interaction networks. More information can be found in the previous discussion and original publications.

**Table 2:** Main features of 20 typical clustering algorithms for extracting clusters from protein interaction networks.

Authors	Methods	Weighted graphs supported	Overlapping clusters supported	Objective	Web-Tool Available
Girvan and Newman 2002 (G-N)	Hierarchical clustering based on betweenness			Functional module	Upon request

Van Dongen S 2000, Enright *et al*.2002 (MCL)	Flow simulation	√		Protein family detection	http://micans.org/mcl/

Spirin and Mirny 2003 (SPC)	Hierarchical	√		Protein complex	http://www.vcclab.org/lab/spc/

Bader and Hogue 2003 (MCODE)	Local neighbourhood density search		√	Protein complex	http://baderlab.org/Software/MCODE

King *et al.* 2004 (RNSC)	Local search cost based			Protein complex	upon request

Radicchi *et al.* 2004 (self contained G-N)	Hierarchical, module definition			Strong module or weak module	upon request

Pržulj *et al.* 2004	Minimum cut (HCS)			Protein complex	upon request

Palla *et al.* 2005 (CPM)	Clique Percolation		√	Protein complex; functional module	http://www.cfinder.org/

Li *et al.* 2005 (LCMA)	Local clique merging		√	Protein complex	upon request

Altaf-UI-Amin *et al.* 2006 (DPClus)	Local density and periphery search		√	Protein complex	http://kanaya.naist.jp/DPClus/

Hwang *et al.* 2006 (STM)	signal transduction		√	Functional module	upon request

Zotenko *et al.* 2006 (COD)	Complex Overlap Decomposition		√	Protein complex	upon request

Luo *et al.* 2007 (MoNet)	Hierarchical, module definition		√	Functional module	upon request

Cho *et al.* 2007 (Semantic integration)	flow-based clustering and Semantic integration	√	√	Functional module	upon request

Ulitsky and Shamir 2007 (MATISSE)	Module Analysis via Topology of Interactions and Similarity	√	√	Functional module	http://acgt.cs.tau.ac.il/matisse/

Gregory 2007 (CONGA)	split betweenness		√	Functional module	upon request

Li *et al.2008* (IPCA)	Local density and distance-based search		√	Protein complex	http://netlab.csu.edu.cn/bioinformatics/limin/IPCA/

Mete *et al.* 2008 (SCAN)	structural clustering based on common neighbors		√	Functional module	upon request

Turanalp and Can 2008 (PPISpan)	gSpan	√	√	Frequent patterns	http://bioserver.ceng.metu.edu.tr/PPISpan/

Li *et al.* 2009 (HC-Wpin)	Hierarchical clustering based on local metric	√		Functional module	http://netlab.csu.edu.cn/bioinformatics/limin/HC-PIN/

## Applications

Typical applications of clustering protein interaction networks are protein function prediction and protein-protein interaction prediction. For a cluster, as pointed by Hartwell *et al.*[106], its members are generally a group of cellular components and their interactions that can be attributed to a specific biological function. Thus, one can identify clusters firstly and then coherently annotate the whole subset of proteins of a given cluster instead of predicting function for individual proteins. Such cluster-assisted methods for predicting protein function differ mainly in their clustering technique. As we have discussed above, distinct clustering results will be obtained by different clustering techniques. After obtaining the clustering result, the methods for protein function prediction are similar. The simplest method is to assign the function shared by the majority of the cluster’s proteins to the function-unknown proteins. Alternatively, a hypergeometric enrichment P-value is calculated for every function of the identified cluster, and the function with the lowest P-value is assigned to the function-unknown proteins.

As there exits a large number of function-unknown proteins, even for the most well-studied yeast, about one-fourth of the proteins remain uncharacterized [117], and the prediction of protein function by laboratory experiments is costly and time consuming, the approaches for predicting protein function based on clustering protein interaction networks are very attractive. Though the prediction can not be the substitute of a lab experiment, it provides references for biologists and experimenters. Moreover, many studies [118,119] have shown that the predictions based on clusters are effective. In a recent review, Sharan *et al.*[117] have given an excellent summary of network-based functional annotation methods and roughly compared direct and cluster-assisted methods for functional annotation. The validation of prediction accuracy highly depends on the knowledge of known annotations. Moreover, the prediction accuracy of the cluster-assisted methods will be affected by the reliabilities of protein interaction networks.

It is well known that the protein-protein interaction data available now are incomplete, though a number of high-throughput biotechnologies have been applied to biological systems. Recently, a series of computational methods have been developed for predicting protein-protein interaction data [120,121]. Especially, the well-developed clustering techniques in protein interaction networks provide new opportunities for completing the protein-protein interaction data. For instances, Yu *et al.*[122] predicted the false negatives based on completing defective cliques, Wang *et al.*[123] suggested an improved method based on maximal cliques for the protein-protein interactions prediction. All these methods are to find highly connected subgraphs in protein interaction networks and to predict the protein-protein interactions based on the supposition that proteins in the same cluster should connect to each other.

**Figure 3:**
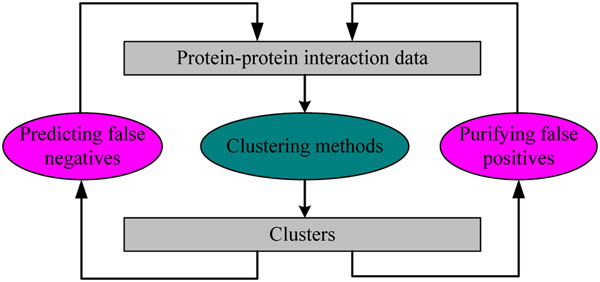
Predicting false negatives and purifying false positives are done on the identified clusters

Clustering protein interaction networks can be used not only for predicting false negatives, but also for purifying false positives, as shown in Fig.3. These two operations: prediction and purification, in turn can also be used as a pre-processing step to improve the accuracy of currently available protein interaction networks.

## Challenges and future researches

In the post-genomic era, an important work is to analyze biological systems from network level, in order to understand the topological organization of protein interaction networks, identify protein complexes and functional modules, discover functions of uncharacterized proteins, and obtain more exact networks. To achieve this aim, a series of clustering approaches have been proposed. For different types of clustering algorithms, each has its own advantages and disadvantages. Every algorithm has certain problems while it exhibits good performances in other cases. The main challenges for clustering protein interaction networks are identified as follows:

(1) Up to now, all methods for predicting protein-protein interactions are known to yield a nonnegligible amount of noise (false positives) and to miss a fraction of existing interactions (false negatives) [10]. Therefore, the protein interaction data available for clustering are very noisy. How to define the quality of a cluster and develop robust algorithm in the presence of noisy edges are challenging.

(2) Clusters of a protein interaction network may overlap with each other. Most proteins have more than one molecular function and participate in more than one biological process. For example, some proteins form transient associations and are part of several complexes at different stages. Most cellular processes are carried out by multi-protein complexes. Therefore, the traditional clustering approaches of putting each protein into one single cluster do not suit this problem well. Moreover, how heavily two clusters should overlap with each other is not certain.

(3) Recent advances in the development of high-throughput techniques have led to an unprecedented amount of protein-protein interaction data becoming available in a variety of simple organisms. It is computationally difficult for most of current clustering algorithms to accurately identify protein complexes or functional modules from large-scale protein interaction networks, especially to discover meso-scale clusters.

(4) There are little priori knowledge for clustering protein interaction networks, such as cluster number and cluster size. How many clusters should we produce? How large are clusters suitable? How to validate different clustering results with various sizes? These are all challenges for designing effective clustering algorithms.

(5) Current clustering approaches mainly focus on detecting clusters in static protein interaction networks for most existing biological data are static. However, both the protein-protein interactions and protein complexes are dynamically organized when implementing special functions. Dynamic modules generally correspond to the sequential ordering of molecular events in cellular systems. How to explore dynamic modules from static protein interaction networks is a very difficult task.

While some clustering approaches have been applied successfully in the discovery of protein complexes or functional modules, methods for clustering and analyzing protein interaction networks are less mature. Particularly, the methods for identifying dynamic modules are in a nascent stage. Methods which use time-series gene expression profiling data to manifest the temporal complexity of protein interaction networks may be useful to the exploration of dynamic modules. For example, Li *et al.*[124] have successfully detected dynamic modules by using the time-series gene expression profiling data. Moreover, spatial constraints [125] may also be an interesting means for further research because proteins belonging to the same functional module should be expressed in the same place.

Furthermore, techniques and methods for developing both robust and fast clustering algorithms are directions for further researches. In the future, “overlap” will continue to be a hot topic for clustering protein interaction networks, which include how many molecular functions a protein can perform, how many biological processes a protein can participate in, and how many cellular components a protein can be associated with or located in. Moreover, we should investigate the question that if there some relationship between the two properties: overlapping and hierarchical organization of clusters, which were usually taken into account separately before. Some works have been done in complex networks, such as word association networks and scientific collaboration networks [86], to detect both the overlapping and hierarchical properties of a community structure. Are the properties also true in protein interaction networks? Additionally, integration of multiple resources will help to detect clusters more accurately and will continue to be interesting.

## Competing interests

The authors declare that they have no competing interests.

## Authors' contributions

JW and ML drafted the manuscript together. YD and YP participated in revising the draft. All authors have read and approved the manuscript.
